# Developments in the Treatment of Leber Hereditary Optic Neuropathy

**DOI:** 10.1007/s11910-022-01246-y

**Published:** 2022-11-21

**Authors:** Benson S. Chen, Patrick Yu-Wai-Man, Nancy J. Newman

**Affiliations:** 1 John Van Geest Centre for Brain Repair and MRC Mitochondrial Biology Unit, Department of Clinical Neurosciences, University of Cambridge, Cambridge, CB2 0PY UK; 2 Cambridge Eye Unit, Addenbrooke’s Hospital, Cambridge University Hospitals, Cambridge, UK; 3 Moorfields Eye Hospital NHS Foundation Trust, London, UK; 4 Institute of Ophthalmology, University College London, London, UK; 5 Department of Ophthalmology, Emory University School of Medicine, Atlanta, GA USA; 6 Department of Neurology, Emory University School of Medicine, Atlanta, GA USA; 7 Department of Neurological Surgery, Emory University School of Medicine, Atlanta, GA USA

**Keywords:** Leber hereditary optic neuropathy, Optic atrophy, Mitochondrial disease, Gene therapy, Idebenone, Allotopic expression

## Abstract

**Purposeof Review:**

To outline the current landscape of treatments for Leber hereditary optic neuropathy (LHON) along the therapeutic delivery pipeline, exploring the mechanisms of action and evidence for these therapeutic approaches.

**Recent Findings:**

Treatments for LHON can be broadly classified as either mutation-specific or mutation-independent. Mutation-specific therapies aim to correct the underlying mutation through the use of a gene-editing platform or replace the faulty mitochondrial DNA-encoded protein by delivering the wild-type gene using a suitable vector. Recent gene therapy clinical trials assessing the efficacy of allotopically expressed *MT-ND4* for the treatment of LHON due to the m.11778G > A mutation in *MT-ND4* have shown positive results when treated within 12 months of symptom onset. Mutation-independent therapies can have various downstream targets that aim to improve mitochondrial respiration, reduce mitochondrial stress, inhibit or delay retinal ganglion cell apoptosis, and/or promote retinal ganglion cell survival. Idebenone, a synthetic hydrosoluble analogue of co-enzyme Q_10_ (ubiquinone), is the only approved treatment for LHON. Mutation-independent approaches to gene therapy under pre-clinical investigation for other neurodegenerative disorders may have the potential to benefit patients with LHON.

**Summary:**

Although approved treatments are presently limited, innovations in gene therapy and editing are driving the expansion of the therapeutic delivery pipeline for LHON.

## Introduction

Leber hereditary optic neuropathy (LHON) is a maternally inherited mitochondrial disorder that presents with severe bilateral sequential vision loss, due to the selective degeneration of retinal ganglion cells (RGCs) [1]. Although a rare condition with an estimated prevalence of 1 in 30,000 to 50,000 in Northern Europe, LHON is the most common cause of inherited mitochondrial blindness globally [2]. Vision loss in LHON is devastating with a significant impact on quality of life [3]. Most patients develop a dense central or caecocentral scotoma in both eyes, and visual acuity is worse than 3/60 (logMAR 1.3), fulfilling the criteria for legal blindness in most countries [4].

Three primary point mutations (m.3460G > A in *MT-ND1*, m.11778G > A in *MT-ND4*, and m.14484 T > C in *MT-ND6*) in the mitochondrial DNA (mtDNA) are responsible for ~ 90% of LHON cases globally [1]. These three primary mutations all involve genes encoding subunits of complex I, the first enzyme of the mitochondrial respiratory chain. In LHON, defective mitochondrial oxidative phosphorylation (OXPHOS) precipitates a bioenergetic crisis that leads to failure of the RGCs. Levels of reactive oxygen species (ROS) also become elevated due to the impaired electron flux along the mitochondrial respiratory chain caused by defective complex I, resulting in oxidative damage to DNA, proteins, and lipids [5]. Increased permeability of the mitochondrial membrane results in instability of mtDNA, further OXPHOS dysfunction, and disrupted Ca^2+^ homeostasis; allowing for the release of signalling factors that eventually trigger cellular apoptosis.

Most individuals who carry a genetic mutation associated with LHON remain asymptomatic. In a national Australian cohort, the penetrance was reported to be 17.5% for males and 5.4% for females [6]. The precise mechanisms that lead to the onset of vision loss remain unknown. External metabolic stressors, such as excessive alcohol consumption and tobacco-smoking, increase the risk of conversion to symptomatic disease. It is hypothesised that external metabolic stressors interfere with normal mitochondrial homeostasis by upsetting the balance between mitochondrial biogenesis and mitophagy [7]. Sex hormones have also been implicated, in particular the protective effects of oestrogens from oxidative stress and the role of testosterone in increasing RGC apoptosis and reduced mitophagy [8, 9].

In most individuals affected by LHON, vision loss due to selective degeneration of RGCs is the only symptom. The RGCs are highly energy-dependent cells that require a constant source of ATP and are sensitive to mitochondrial dysfunction. Dysregulation of superoxide, a specific ROS, appears to be an important cause of aberrant apoptosis signalling in RGCs [10]. Additionally, the unique architecture of RGCs, with an increased concentration of mitochondria in the small calibre, unmyelinated, prelaminar segment of their axons, makes them easily overwhelmed in the setting of impaired mitochondrial biogenesis [11].

Despite the discovery of the mitochondrial genetic basis of LHON by Wallace and colleagues in 1988 [12], there is a paucity of approved effective treatments for LHON. However, substantial progress in understanding the pathogenic mechanisms that culminate in RGC dysfunction and death, as well as the rapid pace of technological innovation within the field of gene therapy and editing, has accelerated the therapeutic delivery pipeline for LHON. In this review, we outline the current landscape of treatments for LHON along the therapeutic delivery pipeline, exploring the mechanisms of action and evidence for these therapeutic approaches. We have provided a glossary of frequently used terms and abbreviations to aid the reader’s understanding (Table 1).

**Table 1:** Glossary of frequently used terms and abbreviations

AAV	Adeno-associated virus; see “viral vector”
Allotopic expression	Expression of genes in the cell nucleus that are normally expressed only by the mitochondrial genome. Requires editing of the mitochondrial DNA into a nuclear-encoded version so that it can be translated and transcribed by the nuclear-cytosolic system that is normally responsible for translating and transcribing nuclear DNA into protein molecules. The edited mitochondrial DNA is delivered to the cell by a viral vector. See also “mitochondrial targeting sequence” and “viral vector”
Complex I	Complex 1 (also known as respiratory complex I; NADH dehydrogenase; and mitochondrial complex I) is the first protein complex of the respiratory chain, responsible for catalysing the transfer of electrons from NADH to co-enzyme Q10 and translocating protons across the inner mitochondrial membrane; see also “OXPHOS”
CRB	Clinically Relevant Benefit; a composite measure of either CRR or CRS being achieved. See “CRR” and “CRS”
CRISPR	Clustered regularly interspaced short palindromic repeats; see “gene editing platform”
CRR	Clinically Relevant Recovery; a measure of improvement in visual acuity. Defined as an improvement from off-chart visual acuity (> 1.68 logMAR) to on-chart visual acuity by at least one full line (+ 5 ETDRS letters) for off-chart eyes OR an improvement in visual acuity by at least two lines (+ 10 ETDRS letters; 0.2 logMAR) for on-chart eyes
CRS	Clinically Relevant Stabilisation; a measure of maintaining visual acuity. Defined as a patient having a visual acuity of < 1.0 logMAR at baseline in at least one eye and maintaining this in the same eye at follow-up
Cybrids	Cybrids (or cytoplasmic hybrids) are cell lines generated by fusing nucleated cells with enucleated cells, resulting in the transfer of cytoplasmic contents of the enucleated cell (such as mtDNA) to the nucleated cell
Gene editing	A group of technologies that enable genetic material to be added, removed, or altered at desired locations in the genome
Gene editing platform	A system containing engineered nucleases or “molecular scissors” that create site-specific breaks at desired locations in the genome. Three distinct classes of nucleases have been discovered and bioengineered to date [see “ZFDs/ZFNs”, “TALE/TALENs”, and “CRISPR”]. Base editing is a newer approach to gene editing that uses components from CRISPR systems together with other enzymes to directly install point mutations into DNA or RNA, without making double-stranded DNA breaks
Mitochondrial heteroplasmy; homoplasmy	A cell can have over 1000 mitochondria, with each mitochondrion containing 2–10 copies of the mitochondrial genome. The mitochondrial genome has a higher mutation rate than the nuclear genome, leading to heterogeneous population of mtDNA within the cell. When two or more different variants of mitochondrial genome coexist within a cell, this is known as heteroplasmy. When all the copies of the mitochondrial genome within a cell are identical, this is known as homoplasmy. Most carriers of a LHON mutation are homoplasmic, i.e. all mitochondria in the retinal ganglion cells will contain the same mitochondrial point mutation
mtDNA	Mitochondrial DNA
*MT-ND1*; *MT-ND4*; and *MT-ND6*	*MT-ND1*; *MT-ND4*; and *MT-ND6* are genes found in the mitochondrial genome that code for subunits of complex I. The three primary LHON mutations are all point mutations involving one of these three genes. See also “complex I”
MTS	Mitochondrial targeting sequence; a targeting peptide chain (sequence of amino acids) that directs the transport of a (newly synthesised) protein to the mitochondria, most often the inner mitochondrial membrane, where the protein complexes for oxidative phosphorylation are located
OXPHOS	Oxidative phosphorylation; the process by which cells produce energy. The process is driven by five protein complexes (complexes I, II, III, IV, and V) located in the inner mitochondrial membrane. See also “complex I”
Penetrance	The proportion of people with a particular genetic mutation (or gene variant) who exhibit signs and symptoms of the genetic disorder
Respiration	see “OXPHOS”
TALE; TALENs	transcription activator-like effector (nucleases); see “gene editing platform”
Viral capsid protein	Capsids are protein shells that surround and protect the viral genome
Viral vector	A modified virus designed to deliver genetic material into cells. AAVs are small viruses that infect humans, but are not known to cause disease except a mild immune response. Several subtypes exist, each with a different tropism (type of cell/tissue they infect)
Viral transduction	the process by which a virus transfers foreign DNA into a cell
ZFDs; ZFNs	Zinc finger deaminases; zinc finger nucleases; see “gene editing platform”

## Therapeutic Development Pipeline

**Fig. 1:**
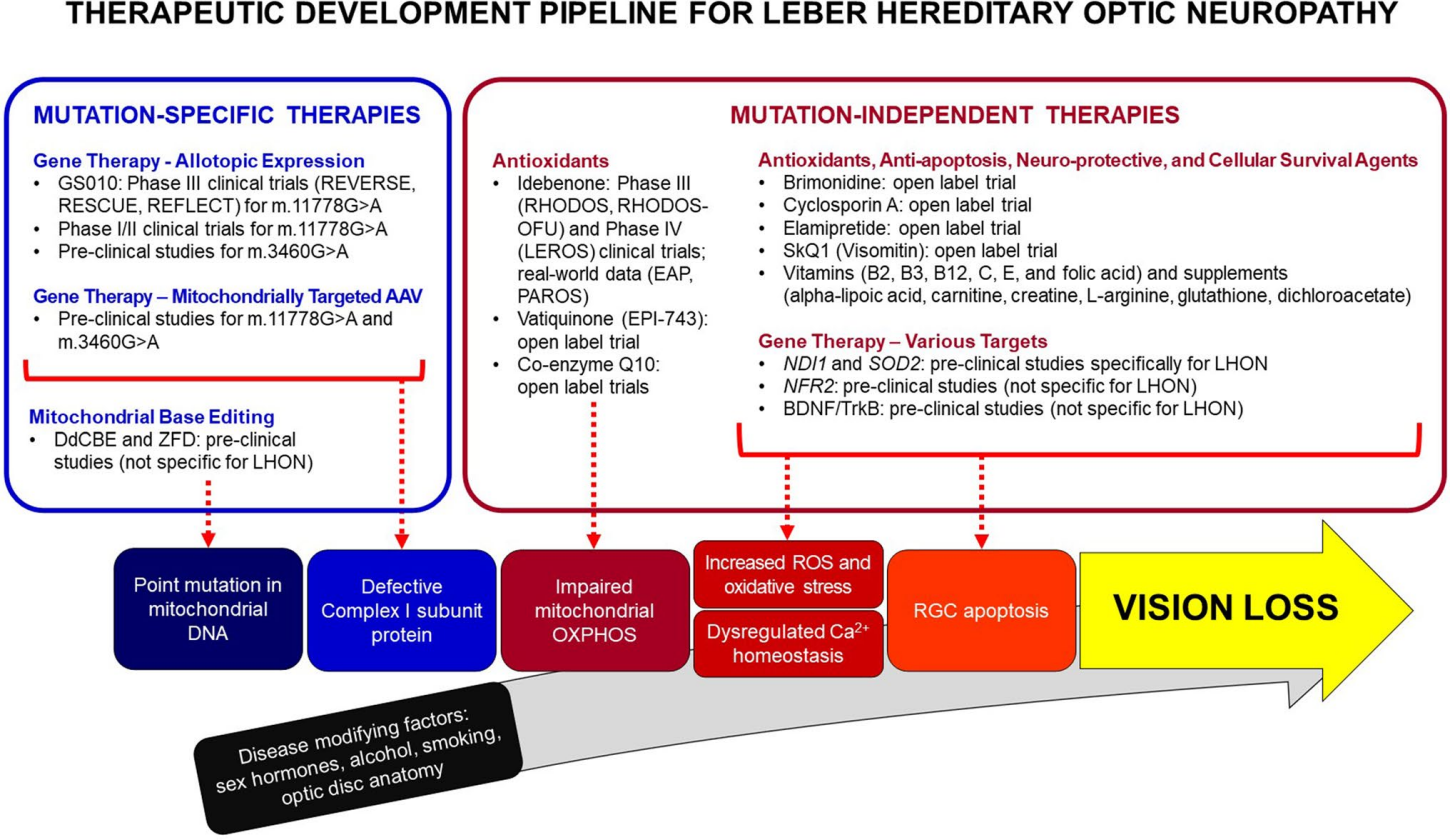
Therapeutic development pipeline for Leber hereditary optic neuropathy (LHON) AAV, adeno-associated virus; BDNF/TrkB, brain-derived neurotrophic factor/tropomycin receptor kinase B; DdCBE, DddA-derived cytosine base editors; EAP, expanded access program; OXPHOS, oxidative phosphorylation; PAROS, post-authorisation observational study; RGC, retinal ganglion cell; ROS, reactive oxygen species; ZFD, zinc finger deaminases

Treatments in the development pipeline for LHON can be broadly divided into two groups based on their therapeutic targets. Therapies can either be mutation-specific or mutation-independent (Fig. 1). Mutation-specific therapies aim to correct the underlying mutation through the use of a gene-editing platform or replace the faulty mtDNA-encoded protein by delivering the wild-type gene using a suitable vector. Mutation-independent therapies can have various downstream targets that aim to improve mitochondrial respiration, reduce mitochondrial stress, inhibit or delay RGC apoptosis, and/or promote RGC survival.

### Mutation-Specific Therapies

The development of gene transfer vectors based on the human adeno-associated virus serotype 2 (AAV2) has revolutionised the treatment of genetic disorders [13]. Several challenges have hampered the development of gene therapy for mitochondrial diseases, most notably the relatively impervious double-membrane structure of mitochondria, which prevents conventional viral vectors from transferring exogenous genes into the mitochondrial genome. For this reason, current approaches to gene therapy in LHON have relied on the technique of allotopic expression.

#### Allotopic Expression

Originally described using yeast in the mid-1980s [14, 15], the technique of allotopic expression was first successfully demonstrated in 2002 in cybrids harbouring the m.11778G > A mutation in *MT-ND4*, by Guy and colleagues at Bascom Palmer Eye Institute in Miami, Florida, USA [16•]. The process of allotopic expression involves delivery of a nuclear-encoded version of the wild-type mitochondrial *MT-ND4* gene into the cell using a viral vector. The gene is expressed in by the nuclear-cytosolic system, and the cytoplasmically-synthesised ND4 protein is imported into the mitochondria with the aid of a mitochondrial targeting sequence (MTS), thereby restoring mitochondrial function. The technique of allotopic expression has also been applied successfully to rescue respiratory chain dysfunction in fibroblasts harbouring the m.3460G > A mutation in *MT*-*ND1* and the development of a gene therapy product “NFS-02” [17, 18]. However, the majority of human clinical trials to date have been conducted for the m.11778G > A mutation, the most prevalent mutation causing LHON globally, with the goal of improving or stabilising vision in patients who have already developed vision loss.

The first human trial of gene therapy for the m.11778G > A mutation was conducted in 2011, in Wuhan, China [19]. Nine patients were treated with a single unilateral IVT injection of their gene therapy product delivered using a recombinant AAV2 vector (“rAAV2-*ND4*”). Long-term follow-up data was reported for eight participants [20]. Despite unilateral treatment, there was a bilateral improvement in best-corrected visual acuity (BCVA) in five patients at 7.5 years post-treatment. “Improvement” was defined as an increase in BCVA of at least 0.3 logMAR from baseline. Improvement mostly occurred 3–6 months post-treatment and persisted through the follow-up period, with treated eyes (the eye receiving the IVT injection) showing greater improvement compared to the untreated eye [19, 21]. Greater visual recovery occurred in younger patients and those treated within 1–2 years of disease onset. In a second study, a separate cohort of 149 patients with mean disease duration of 40.56 months (SD 49.99, range 1–312 months) received a single unilateral IVT injection [22]. In this cohort, patient age, the period between onset and treatment, and pre-treatment BCVA, were identified as important predictors of rapid and significant improvement after treatment. Additional Phase I/II clinical trials are being planned by a spin-off company, Neurophth Therapeutics (Wuhan, China).

The group at Bascom Palmer conducted a Phase I single-centre, open-label, dose-escalation study of their gene therapy product (“scAAV2(Y444,500,730F)-*P1ND4*v2”) [23–25]. Twenty-eight participants were treated with a single unilateral IVT injection in four escalating doses, including 17 participants with a disease duration up to 1 year (eight of whom had unilateral disease) and 11 participants with chronic bilateral disease with a duration more than 1 year. Bilateral improvement was also observed, with greater improvement occurring in the treated eye [23, 24]. However, there was no noteworthy change in BCVA in patients with chronic disease at 36 months follow-up when compared to a natural history cohort [25]. Similarly, improvement in treated and untreated eyes of nine patients with bilateral vision loss (duration up to one year) was not significant when compared to the spontaneous improvement that occurred in nine control patients. The investigators concluded that the efficacy of gene therapy was likely to be small and not dose-related [25]. Finally, this study investigated the effects of injecting the unaffected eye in patients with unilateral disease and found that gene therapy failed to prevent vision loss within the first year after treatment. The main limitation of this study was the small participant number. Treated eyes tended to show more improvement from 24 months onwards. However, there were fewer control patients at 24 and 36 months follow-up, thereby reducing the study’s ability to accurately determine treatment effect. It is unknown whether further clinical trials are planned for this gene therapy product, especially due to the success of other gene therapy products for the m.11778G > A mutation in *MT-ND4*.

Three Phase III multi-centre, randomised, double-masked, sham- or placebo-controlled clinical trials (“REVERSE” [26], “RESCUE” [27], and “REFLECT” [28]) were conducted following an initial Phase I/IIa open-label, single-centre, dose-escalation clinical trial (“REVEAL” [29, 30]) of rAAV2/2-*ND4* (GS010, lenadogene nolparvovec). In the REVERSE and RESCUE trials, 37 participants with bilateral vision loss duration of 6–12 months (REVERSE) and 38 participants with bilateral vision loss duration of ≤ 6 months (RESCUE) were treated with a unilateral IVT injection. Participants in the REVERSE study exhibited bilateral improvements in BCVA, contrast sensitivity, and on automated perimetry. At week 96 post-injection, the mean change in logMAR acuity from baseline was − 0.31 (equivalent to + 15 ETDRS letters) in treated eyes and − 0.60 (+ 13 ETDRS letters) in sham-treated eyes [26]. Despite earlier treatment and better baseline BCVA in the RESCUE trial, visual outcomes at week 96 were actually somewhat inferior to those seen in REVERSE [27]. Participants in RESCUE had deterioration of vision in both eyes initially, reaching a nadir around week 24. At week 96 post-injection, the mean change from baseline was + 0.18 (− 9 ETDRS letters) in treated eyes and + 0.21 (− 10 ETDRS letters) in sham-treated eyes [27]. The investigators speculated that axonal swelling in the initial stages of the disease could play a role by imposing a physical barrier to the diffusion of rAAV2/2-*ND4* to RGCs and potentially impede the distribution of the viral vector throughout the retinal nerve fibre layer after viral transduction.

Participants of the REVERSE and RESCUE studies were followed for an additional 3 years (corresponding to 5 years post-IVT injection) as part of a long-term follow-up study (“RESTORE” [31]). To account for differences in the duration of vision loss at the time gene therapy was administered, a locally estimated scatterplot smoothing (LOESS) nonparametric local regression model was used to independently assess each patient’s eyes beginning 12 months after the onset of vision loss, when 92.7% of eyes (139/150) had received treatment. The LOESS regression model showed a progressive and sustained improvement in BCVA from 12 to 51.5 months after the onset of vision loss [31]. Compared to a natural history cohort comprising patients pooled from 11 natural history studies, there was a statistically and clinically relevant difference in BCVA of − 0.33 (+ 16.5 ETDRS) in favour of treated eyes at 48 months after the onset of vision loss (*p* < 0.01) [32•]. Most treated eyes (88.7%) had BCVA better than 1.6 logMAR at month 48 compared to 48.1% of natural history eyes (*p* < 0.01). Importantly, the treatment effect was durable, with BCVA at the last follow-up remaining statistically and clinically significant when adjusted for age and duration of follow-up.

In the most recent REFLECT study, 98 participants, with bilateral vision loss duration ≤ 1 year from the onset, received an IVT injection of rAAV2/2-*ND4* in their first affected eye, and either a second IVT injection of rAAV2/2-*ND4* or a placebo in their second affected eye [28]. There was a trend towards slightly better visual improvement with bilateral treatment. At 2 years post-IVT injection, the mean improvement in BCVA of bilaterally treated eyes was -0.39 (+ 20 ETDRS letters) and − 0.34 (+ 17 ETDRS letters) in the first and second treated eyes, respectively, compared to nadir. Both eyes in unilaterally treated patients also improved significantly compared to nadir, with a − 0.38 (+ 19 ETDRS letters) and − 0.27 (+ 14 ETDRS letters) improvement observed in treated and placebo-treated eyes, respectively [21].

Although the recent clinical trials have demonstrated the potential benefits of gene therapy for the m.11778G > A mutation using the technique of allotopic expression, GS010 remains an experimental therapy that has not yet been evaluated by the US Food and Drug Administration (FDA) and the European Medicine Agency (EMA). This has been partly due to the unexpected observation of bilateral visual improvement across all gene therapy studies conducted with unilateral IVT injection and the lack of a patient placebo-controlled arm in each of these trials. Spontaneous improvement in vision is known to occur as part of the natural history of LHON. However, the improvement achieved with gene therapy significantly differs from the published natural history of LHON, where an ultimate visual acuity of better than 1.0 logMAR is rare [32•, 33••]. The underlying mechanisms of possible inter-eye transfer of the rAAV2/2-*ND4* viral vector have been investigated in non-human primates and transneuronal spread via the optic nerve and chiasm by synaptic transfer mechanisms has been put forward as a potential route to explain the demonstrated presence of viral vector within the contralateral untreated retina and optic nerve [26].

Although it has been suggested that further clinical trials with a patient placebo-controlled arm are required to truly confirm the efficacy of this gene therapy, this can be challenging in rare diseases like LHON. The FDA does recognise that in some studies it is not feasible or ethical to use an “internal control” and reliance on an “external control” is acceptable [34]. Indeed, there is precedent for regulatory approval without an internal control, with at least 45 drugs and biologic products approved by the FDA previously, using external control data in their risk–benefit assessment, with reasons including the rare nature of the disease; ethical concerns regarding the use of a placebo or no-treatment arm; the seriousness of the condition; and high unmet medical need [34]. However, in January 2022, the FDA recommended that GenSight Biologics “conduct an additional placebo-controlled trial to bolster the demonstration of [GS010] efficacy in view of the unexpected bilateral effect observed in unilaterally treated patients in RESCUE, REVERSE, and REFLECT trials” [35]. GenSight Biologics submitted a marketing authorisation application for GS010 to the EMA in September 2020 and, in a press release, stated that “[they] expect[s] an opinion from the EMA’s Committee for Medical Products for Human Use (CHMP) by Q3 2023, to be followed by commercial launch by the end of 2023” [36].

The results of the Phase III studies only provide evidence for treating individuals within 12 months of symptom onset. The evidence for gene therapy in chronic LHON (> 12 months from symptom onset) remains unclear. A clinical trial (“REVIVE”) to study the efficacy of GS010 in patients with chronic LHON, more than 1 year but no more than 5 years from the onset of symptoms, will commence in 2023 to address this issue [37]. Patient recruitment for REVIVE will be limited to residents of the UK.

#### Mitochondrially-Targeted Adeno-Associated Viral Vectors

Another approach to gene therapy under pre-clinical investigation is the use of a modified AAV, where one of the viral capsid proteins is modified to include an MTS (“MTS-AAV”). Similar to the MTS that directs the cytoplasmically synthesised protein to the mitochondria, the MTS directs the AAV to the mitochondria where the packaged gene can be delivered. The MTS-AAV has been successfully employed to deliver the human mutant *ND4* gene in a mouse model that recapitulates the hallmarks of human LHON [38]; deliver wild-type human *ND4* gene to rescue the defective respiration of cybrids carrying the m.11778G > A mutation [39]; and deliver human mutant and wild-type *ND1* gene in a mouse model that recapitulates the hallmarks of human LHON and rescues defective complex I-dependent production of ATP [40]. The MTS-AAV approach may be more efficient than the technique of allotopic expression, as the wild-type version of the mutated mtDNA gene does not require modification to the nuclear node, nor is a separate import and assembly pathway required. The group at Bascom Palmer is performing pre-clinical testing of MTS-AAV-delivered *ND1* to gain regulatory approval of this therapy for human testing [40].

#### Gene Editing

Recent advances in gene editing techniques offer alternative approaches to gene therapy for LHON [41, 42•]. Although gene editing with clustered regularly interspaced short palindromic repeats (CRISPR) systems has been discussed as a revolutionary tool for gene editing, current CRISPR systems that utilise a guide RNA are ineffective because mitochondria do not readily import RNA [43].

One approach to gene editing is the use of mitochondrially targeted zinc finger nucleases (ZFNs) and transcription activator-like effector nucleases (TALENs) [38]. Both mitochondrially targeted ZFNs and TALENs are composed of two functional units: a component that recognises a specific DNA sequence and a component that mediates DNA cutting. By cutting the segment of mtDNA that contains the point-mutation causing disease, this approach aims to shift mtDNA heteroplasmy, i.e. lower the level of the pathogenic mtDNA variant [44, 45]. Successful proof-of-principle experiments have been conducted in cybrids containing pathogenic variants that cause neuropathy, ataxia, and retinitis pigmentosa (NARP) [44]. However, as most LHON carriers are homoplasmic (i.e. all copies of the mitochondrial genome carry the pathogenic variant), ZFN and TALENs have limited applications for the treatment of LHON. One potential application is the utilisation of ZFNs and TALENs to induce heteroplasmy shift in oocytes or embryos containing high levels of the pathogenic mtDNA variant prior to implantation, thereby preventing transmission of LHON to the next generation [46].

Mitochondrial base editing is now a reality with the use of a cytosine base editor (DdCBE), which combines a transcription activator-like effector (TALE) array with a double-stranded DNA-specific cytidine deaminase (DddA), to mediate targeted C•G-to-T•A editing [47•, 48]. Another mitochondrial base editing platform is zinc finger deaminases (ZFDs), which contain a zinc-finger binding protein instead of a TALE [49]. Both DdCBE and ZFDs platforms are still in their infancy and limited to C•G-to-T•A editing. Further pre-clinical studies are required to determine if either technique is a viable strategy for the treatment of LHON due to the m.14484 T > C mutation, the only primary LHON mutation that would be amenable to this therapeutic approach.

### Mutation-Independent Therapies

Mutation-independent therapies for LHON aim to improve mitochondrial respiration, reduce mitochondrial stress, inhibit or delay RGC apoptosis, and promote RGC survival, without correcting the underlying genetic defect. These therapies are attractive as they can be utilised in patients for which a mutation-specific therapy currently does not exist or is unlikely to be developed.

Several antioxidants, including different vitamins (B2, B3, B12, C, E, and folic acid) and supplements (alpha-lipoic acid, carnitine, creatine, L-arginine, glutathione, and dichloroacetate) have been investigated specifically for use in LHON, without clear demonstration of clinical benefit [50]. SkQ1 (tradename Visomitin), a synthetic “mitochondrial-targeted antioxidant” was recently reported to improve BCVA by 0.37 logMAR in 26 LHON patients receiving the drug for dry eyes, after 12 months treatment [51]. However, key clinical details such as the duration of vision loss, the age of participants, and baseline BCVA were not reported.

Clinical studies have also been conducted for brimonidine, cyclosporine A, and elamipretide (MTP-131). Neither brimonidine, an α-2 agonist with potent neuroprotective effects in experimental animal models of optic nerve injury, or cyclosporine A, a potent immunomodulatory agent that has a crucial role in blocking apoptosis in damage-induced cell death, prevented second eye involvement in two separate open-label trials focusing on patients with acute LHON who were still unilaterally affected [52, 53]. In a Phase II double-masked trial, there was no difference in BCVA at 84 weeks in patients with chronic LHON treated with topical elamipretide, a mitochondria-targeting peptide that promotes increased ATP production and decreased ROS production [54].

The ubiquinone family, in particular idebenone, has shown the most promise and will be discussed in detail. Mutation-independent therapies that utilise AAV vectors to deliver specific genes associated with cell survival and mitochondrial regulation have also been under investigation and are discussed below.

#### Idebenone

Co-enzyme Q_10_, also known as ubiquinone, plays an important role in the mitochondrial respiratory chain by carrying electrons from complexes I and II to complex III. Co-enzyme Q_10_ has been beneficial for some inherited mitochondrial disorders. However, administration of oral CoQ_10_ has not been shown to be beneficial in LHON, presumably because of its inability to cross the blood–brain barrier. To overcome this issue, idebenone, a synthetic hydrosoluble analogue of CoQ_10_ was developed. In preclinical studies, idebenone partially restores cellular ATP levels under conditions of impaired complex I function [55]. In patients with LHON, idebenone is thought to transfer electrons directly to complex III, thereby providing a redox bypass of complex I [56].

In the first clinical trial of oral idebenone, Rescue of Hereditary Optic Disease Outpatient Study (“RHODOS”), 85 patients with LHON with < 5 years of visual loss were randomised to receive either 900 mg/day idebenone or a placebo for 24 weeks [57]. At week 24, idebenone was associated with better BCVA than placebo in the intention-to-treat population. However, the primary endpoint of difference in best recovery in visual acuity did not reach statistical significance [− 0.064 logMAR; 95% CI − 0.184 to + 0.055; *P* = 0.291). Excluding participants who carried the m.14484 T > C mutation, which is known for its higher rate of spontaneous improvement in visual acuity, led to a larger treatment effect, which still remained non-significant [57].

In a follow-up study (“RHODOS-OFU”), RHODOS participants were assessed a median of 30 months after they had discontinued idebenone and placebo [58]. The mean difference in the best recovery of visual acuity between treatment groups for the entire period from baseline of RHODOS to the RHODOS–OFU visit was − 0.158 logMAR (+ 7 ETDRS, *P* = 0.086), in favour of idebenone. When mean change in visual acuity of individual eyes from baseline RHODOS to RHODOS-OFU was analysed, a significant improvement in visual acuity was detected in participants treated with idebenone (− 0.228; + 11 letters; *P* = 0.0011). The treatment effect was greater (− 0.283; + 14 letters, *P* = 0.0002) for patients carrying either the m.11778G > A or m.3460G > A mutation, after those with the m.14484 T > C mutation were excluded from the analysis [58]. A retrospective observational study of patients receiving idebenone found that patients were more likely to recover vision if treatment was initiated early and if it was maintained for longer than the 24 weeks regimen used in RHODOS [59]. The findings of this study supported the hypothesis that idebenone has the highest impact if initiated early in the disease at a time when RGC loss is minimal and there are still viable, but inactive, RGCs [58, 59].

The timing of initiating treatment and the duration of treatment were further explored in a real-world observational study of patients taking idebenone as part of an expanded access programme (EAP) setup by the drug manufacturer [60•]. A total of 87 patients were included in the analysis. All carried one of the three primary LHON mutations and had started treatment within 12 months of symptom onset, with the dose and duration of treatment at the discretion of the treating physician. Mean treatment duration was 25.6 months (2.4–70.4 months). A clinically relevant recovery (CRR) of BCVA, defined as an improvement from off-chart acuity (> 1.68 logMAR) to on-chart by at least one full line (+ 5 ETDRS letters) or an improvement in an on-chart BCVA by at least two lines (+ 10 ETDRS letters; 0.2 logMAR), was observed in 40 (46.0%) patients. The proportion of patients with recovery and the magnitude of recovery increased with treatment duration. The investigators concluded that a treatment duration of at least 18–24 months was needed to maximise the probability of CRR, particularly as 33% of patients who experienced CRR did so after 12 months of treatment [60•].

In 2015, the EMA granted idebenone orphan medicine status and authorised its use under exceptional circumstances [61]. To address issues regarding the duration and timing of initiation of treatment, a long-term Phase IV externally-controlled open-label study (“LEROS”) was designed with guidance from the EMA. A total of 199 patients with one of the three primary mutations of LHON commenced idebenone within 5 years of symptom onset and were treated for up to 24 months [62]. The primary endpoint was the proportion of participants experiencing a clinically relevant benefit (CRB), defined as either a CRR or a clinically relevant stabilisation (CRS) of BCVA (maintenance of BCVA < 1.0 logMAR) or both. In patients treated within 12 months of symptom onset, 42.3% (60/142) of treated eyes experienced a CRB at 12 months, compared to 20.7% (40/193) in a matched external natural history cohort (*P* = 0.002) [62]. This difference was maintained after 24 months (52.9% [64/121] vs 36.0% [27/75], *P* = 0.0297]). After 24 months treatment, participants had a median BCVA of 1.07 logMAR, compared to 1.28 logMAR at baseline. Positive results were also seen in participants who commenced idebenone > 12 months after symptom onset compared to the natural history group. In chronic eyes, CRB was observed in 50.3% (72/143 eyes) vs 38.6% (59/153) from the natural history cohort at 12 months (*P* = 0.0087); and 49.1% (57/116) vs 37.6% (35/93) at 24 months (*P* = 0.0175) [63].

Taken together, the body of evidence to date indicates that idebenone appears to be efficacious in improving visual function or stabilising vision in individuals with LHON. Early initiation of treatment after the onset of vision loss and a longer treatment duration appears to be associated with better odds of improving or stabilising vision. According to the 2017 “International Consensus Statement on the Clinical and Therapeutic Management of Leber Hereditary Optic Neuropathy” [64••], idebenone should be started as soon as possible at 900 mg/day in patients with disease less than 1 year, and treatment continued for at least 1 year to assess response. If CRR is confirmed, treatment should be continued for another 1 year. The recent results from the LEROS study indicate that patients with chronic LHON treated within 5 years of symptom onset also have the potential to benefit from idebenone. Another study, the Post-Authorisation Safety Study with Raxone (“PAROS”), is currently in progress with the objective of determining the long-term safety and effectiveness of idebenone when used under conditions of routine care. Patient access to idebenone remains an area of concern. Outside Europe, idebenone is approved for use in Israel but has not received regulatory approval in the USA, Canada, Australia, or New Zealand. In the UK, idebenone is approved and funded on the pharmaceutical schedule in Northern Ireland, Scotland, and Wales, but not England. Decisions regarding regulatory approval and funding in some countries used only data from the RHODOS study, which was limited by a short treatment duration and follow-up. Data from recent studies, in particular LEROS, provide a more accurate picture of the efficacy of idebenone in a subset of patients with LHON, in particular those with chronic LHON between 1 and 5 years disease duration.

The efficacy of idebenone in patients with chronic LHON > 5 years duration is questionable, due to their exclusion in the RHODOS and LEROS trials. A small retrospective observational study of seven patients receiving treatment with idebenone > 5 years after symptom onset found that BCVA improved significantly by a mean of − 0.20 logMAR (+ 10 ETDRS) (*P* =  − 0.002) in the first year of treatment [65]. However, the main limitation of the study was the selection of participants, four of whom harboured the m.14484 T > C mutation. The study’s authors hypothesised that the treatment response was the result of reactivated signal transduction in surviving dysfunctional RGCs. Additionally, idebenone is not authorised for use in asymptomatic individuals. However, given that visual stabilisation was observed in a proportion of participants in the LEROS study, it may be useful for future studies to explore whether idebenone modifies the risk of developing vision loss by increasing mitochondrial reserve, especially in individuals who would be at higher risk such as males or those with exposure to external metabolic stressors that disrupt OXPHOS.

#### Vatiquinone

Vatiquinone (EPI-743) is a quinone molecule derived from the hydrolysis of vitamin E. Initially developed for the treatment of Leigh syndrome, vatiquinone has been investigated in several clinical trials for inherited mitochondrial diseases and neurodegenerative diseases including for Friedreich ataxia, where it has been granted Orphan Drug Designation and Fast Track Designation by the FDA [66]. Vatiquinone crosses the blood–brain barrier and is approximately 1000-to 10,000-fold more potent than CoQ_10_ or idebenone in protecting mitochondrial patient fibroblasts in oxidative stress assays [67]. The efficacy of vatiquinone has only been reported in small case series, with reports of improved vision, including reversal of visual loss, in patients treated within 90 days of acute vision loss [68–70]. Although results are favourable when compared to a natural history cohort, an adequately powered clinical trial is needed to further explore its efficacy.

#### Mutation-Independent Approaches to Gene Therapy

Adeno-associated viral vectors have been used to deliver genes that improve defective mitochondrial function and increase RGC survival. Specific genes that have been investigated previously include *NDI1, SOD2*, and *NRF2*.

Ndi1, encoded by the *NDI1* gene, is an alternative NADH dehydrogenase expressed in yeast mitochondria (equivalent to mammalian complex I). In a rotenone-induced murine model of LHON, optic nerve degeneration was rescued by delivering yeast *NDI1* [71, 72]. The expressed protein product, Ndi1, acted as a functional replacement for defective complex I, restoring electron transfer and suppressing ROS production.

Superoxide dismutase, encoded by the *SOD2* gene, catalyses the dismutation of superoxide radicals in mitochondria and is a key mediator of the cell’s antioxidant defence mechanism. In a study of m.11778G > A LHON cybrids transfected with an AAV vector encoding the *SOD2* gene, overexpression of superoxide dismutase resulted in increased cell survival [73]. Similarly, in a study of HEK293T cybrids subjected to oxidative stress, overexpression of Nrf2 (encoded by the *NRF2* gene) resulted in a reduction of ROS levels and increased cell survival [74]. Nrf2 is a transcription factor involved in mitochondrial biogenesis and is a major regulator of antioxidant and cellular protective genes, including *SOD2*.

Another target for gene expression that is currently under investigation in models of neurodegeneration, but with potential application in LHON, is the brain-derived neurotrophic factor (BDNF*)*/tropomycin receptor kinase B (TrkB) signalling pathway. In experimental models of glaucoma, overexpression of BDNF alleviated RGC loss after optic nerve injury and improved RGC survival, but this effect is time-limited because of downregulation of TrkB (the BDNF receptor) [75]. Recent studies have shown that gene therapy that delivers both BDNF and TrKB is more effective than gene therapy with BDNF or TrKB alone [76]. Combined overexpression of BDNF and TrKB was effective in stimulating axon transport in an experimental glaucoma model [76]. Treated RGCs exhibited functional recovery, determined by the RGC-specific positive scotopic threshold response.

Although a mitochondrial disorder, LHON shares a final common pathway as in other neurodegenerative disorders, namely (RGC-specific) neuronal degeneration and disrupted axonal transport. Development of therapies for other conditions that share this final common pathway could benefit patients with LHON. However, further studies are required to determine the safety of these approaches, in particular cross-species approaches to gene therapy such as delivery of *NDI1*. Furthermore, the effects of multiple treatments with AAV2 vectors and the development of neutralising antibodies remain unknown, especially in patients who have already received a mutation-specific gene therapy [77].

## Conclusion

The therapeutic development pipeline for LHON has experienced rapid growth over the past decade, driven by innovations in gene therapy and editing. Although large clinical trials have focused on gene therapy for the m.11778G > A LHON mutation using the technique of allotopic expression, considerable progress has also been made in the development of mutation-independent therapies that aim to improve mitochondrial function and enhance RGC survival. The development of new gene editing platforms may offer solutions that are currently thought to be impossible. The clinical trials for GS010 (REVERSE, RESCUE, REFLECT) and idebenone (RHODOS, RHODOS-OFU, LEROS) highlight some of the challenges of conducting clinical trials in individuals with rare diseases, emphasising the importance of collaborative research and careful clinical trial design, in particular the selection of outcome measures. The lessons learned from these trials need to be applied to future trials, in order to maximise the success of new treatments progressing through the therapeutic development pipeline, from bench to bedside. It is clear that LHON is a complex multifactorial disease and a multimodal approach to treatment may be required given the rapid catastrophic loss of RGCs that occurs in the acute stage of this mitochondrial genetic disorder.

## References

[1] Yu-Wai-Man P, Griffiths PG, Chinnery PF (2011). Mitochondrial optic neuropathies - disease mechanisms and therapeutic strategies. Prog Retin Eye Res.

[2] Yu-Wai-Man P, Griffiths PG, Brown DT, Howell N, Turnbull DM, Chinnery PF (2003). The epidemiology of Leber hereditary optic neuropathy in the North East of England. Am J Hum Genet.

[3] Chen BS, Holzinger E, Taiel M, Yu-Wai-Man P (2022). The impact of Leber hereditary optic neuropathy on the quality of life of patients and their relatives: a qualitative study. J Neuroophthalmol.

[4] Yu-Wai-Man P, Newman NJ, Carelli V, La Morgia C, Biousse V, Bandello FM (2022). Natural history of patients with Leber hereditary optic neuropathy-results from the REALITY study. Eye (Lond).

[5] Yang TC, Yarmishyn AA, Yang YP, Lu PC, Chou SJ, Wang ML (2020). Mitochondrial transport mediates survival of retinal ganglion cells in affected LHON patients. Hum Mol Genet.

[6] Lopez Sanchez MIG, Kearns LS, Staffieri SE, Clarke L, McGuinness MB, Meteoukki W (2021). Establishing risk of vision loss in Leber hereditary optic neuropathy. Am J Hum Genet.

[7] Mejia-Vergara AJ, Seleme N, Sadun AA, Karanjia R (2020). Pathophysiology of conversion to symptomatic Leber hereditary optic neuropathy and therapeutic implications: a review. Curr Neurol Neurosci Rep.

[8] Jankauskaite E, Ambroziak AM, Hajieva P, Oldak M, Tonska K, Korwin M (2020). Testosterone increases apoptotic cell death and decreases mitophagy in Leber’s hereditary optic neuropathy cells. J Appl Genet.

[9] Pisano A, Preziuso C, Iommarini L, Perli E, Grazioli P, Campese AF (2015). Targeting estrogen receptor beta as preventive therapeutic strategy for Leber’s hereditary optic neuropathy. Hum Mol Genet.

[10] Levin LA (2007). Mechanisms of retinal ganglion specific-cell death in Leber hereditary optic neuropathy. Trans Am Ophthalmol Soc.

[11] Yu Wai Man CY, Chinnery PF, Griffiths PG (2005). Optic neuropathies-importance of spatial distribution of mitochondria as well as function. Med Hypotheses.

[12] Wallace DC, Singh G, Lott MT, Hodge JA, Schurr TG, Lezza AM (1988). Mitochondrial DNA mutation associated with Leber’s hereditary optic neuropathy. Sci.

[13] Dunbar CE, High KA, Joung JK, Kohn DB, Ozawa K, Sadelain M. Gene therapy comes of age. Science. 2018;359:eaan4672. 10.1126/science.aan4672 10.1126/science.aan4672 29326244

[14] Gearing DP, McMullen GL, Nagley P (1985). Chemical synthesis of a mitochondrial gene designed for expression in the yeast nucleus. Biochem Int.

[15] Gearing DP, Nagley P (1986). Yeast mitochondrial ATPase subunit 8, normally a mitochondrial gene product, expressed in vitro and imported back into the organelle. EMBO J.

[16] Guy J, Qi X, Pallotti F, Schon EA, Manfredi G, Carelli V (2002). Rescue of a mitochondrial deficiency causing Leber hereditary optic neuropathy. Ann Neurol.

[17] Bonnet C, Augustin S, Ellouze S, Benit P, Bouaita A, Rustin P (2008). The optimized allotopic expression of ND1 or ND4 genes restores respiratory chain complex I activity in fibroblasts harboring mutations in these genes. Biochim Biophys Acta.

[18] Bonnet C, Kaltimbacher V, Ellouze S, Augustin S, Benit P, Forster V (2007). Allotopic mRNA localization to the mitochondrial surface rescues respiratory chain defects in fibroblasts harboring mitochondrial DNA mutations affecting complex I or v subunits. Rejuvenation Res.

[19] Wan X, Pei H, Zhao MJ, Yang S, Hu WK, He H (2016). Efficacy and safety of rAAV2-ND4 treatment for Leber’s hereditary optic neuropathy. Sci Rep.

[20] Yuan J, Zhang Y, Liu H, Wang D, Du Y, Tian Z (2020). Seven-year follow-up of gene therapy for Leber’s hereditary optic neuropathy. Ophthalmol.

[21] Yang S, Ma SQ, Wan X, He H, Pei H, Zhao MJ (2016). Long-term outcomes of gene therapy for the treatment of Leber’s hereditary optic neuropathy. EBioMed.

[22] Liu HL, Yuan JJ, Zhang Y, Tian Z, Li X, Wang D (2020). Factors associated with rapid improvement in visual acuity in patients with Leber’s hereditary optic neuropathy after gene therapy. Acta Ophthalmol.

[23] Feuer WJ, Schiffman JC, Davis JL, Porciatti V, Gonzalez P, Koilkonda RD (2016). Gene therapy for Leber hereditary optic neuropathy: initial results. Ophthalmol.

[24] Guy J, Feuer WJ, Davis JL, Porciatti V, Gonzalez PJ, Koilkonda RD (2017). Gene therapy for Leber hereditary optic neuropathy: low- and medium-dose visual results. Ophthalmol.

[25] Lam BL, Feuer WJ, Davis JL, Porciatti V, Yu H, Levy RB (2022). Leber hereditary optic neuropathy gene therapy: adverse events and visual acuity results of all patient groups. Am J Ophthalmol.

[26] Yu-Wai-Man P, Newman NJ, Carelli V, Moster ML, Biousse V, Sadun AA (2020). Bilateral visual improvement with unilateral gene therapy injection for Leber hereditary optic neuropathy. Sci Transl Med.

[27] Newman NJ, Yu-Wai-Man P, Carelli V, Moster ML, Biousse V, Vignal-Clermont C (2021). Efficacy and safety of intravitreal gene therapy for Leber hereditary optic neuropathy treated within 6 months of disease onset. Ophthalmol.

[28] Newman NJ, Yu-Wai-Man P, Subramanian PS, Moster ML, Wang AG, Donahue SP, Leroy BP, Carelli V, Biousse V, Vignal-Clermont C, Sergott RC, Sadun AA, Fernández GR, Chwalisz BK, Banik R, Bazin F, Roux M, Cox ED, Taiel M, Sahel JA; LHON REFLECT Study Group. Randomized trial of bilateral gene therapy injection for m.11778G > A MT-ND4 Leber optic neuropathy. Brain. 2022;awac421. 10.1093/brain/awac421. 10.1093/brain/awac421 PMC10115230 36350566

[29] Vignal C, Uretsky S, Fitoussi S, Galy A, Blouin L, Girmens JF (2018). Safety of rAAV2/2-ND4 gene therapy for Leber hereditary optic neuropathy. Ophthalmol.

[30] Vignal-Clermont C, Girmens JF, Audo I, Said SM, Errera MH, Plaine L (2021). Safety of intravitreal gene therapy for treatment of subjects with Leber hereditary optic neuropathy due to mutations in the mitochondrial ND4 gene: the REVEAL study. BioDrugs.

[31] Biousse V, Newman NJ, Yu-Wai-Man P, Carelli V, Moster ML, Vignal-Clermont C (2021). Long-term follow-up after unilateral intravitreal gene therapy for Leber hereditary optic neuropathy: the RESTORE study. J Neuroophthalmol.

[32] Newman NJ, Yu-Wai-Man P, Carelli V, Biousse V, Moster ML, Vignal-Clermont C (2021). Intravitreal gene therapy vs. natural history in patients with Leber hereditary optic neuropathy carrying the m.11778G>A ND4 mutation: systematic review and indirect comparison. Front Neurol.

[33] Newman NJ, Carelli V, Taiel M, Yu-Wai-Man P (2020). Visual outcomes in Leber hereditary optic neuropathy patients with the m.11778G>A (MTND4) mitochondrial DNA mutation. J Neuroophthalmol.

[34] Jahanshahi M, Gregg K, Davis G, Ndu A, Miller V, Vockley J (2021). The use of external controls in FDA regulatory decision making. Ther Innov Regul Sci.

[35] GenSight Biologics. GenSight Biologics Reports Cash Position as of December 31, 2021, and Provides Operational Update [Internet]. Paris, France: GenSight Biologics; 18 January 2022 [cited 1 November 2022]. Available from: https://www.gensight-biologics.com/2022/01/18/gensight-biologics-reports-cash-position-as-of-december-31-2021-and-provides-operational-update/

[36] GenSight Biologics. GenSight Biologics Reports Cash Position and Revenues as of September 30, 2022 [Internet]. Paris, France: GenSight Biologics; 28 October 2022 [cited 1 November 2022]. Available from: https://www.gensight-biologics.com/2022/10/28/gensight-biologics-reports-cash-position-and-revenues-as-of-september-30-2022/

[37] Jackson J. Gene therapy trial aims to help restore sight for LHON patients [Internet]. Manchester, United Kingdom: National Health Executive; 2 September 2021 [cited 10 July 2022]. Available from: https://www.nationalhealthexecutive.com/articles/new-gene-therapy-trial-restore-sight-lhon-patients

[38] Yu H, Ozdemir SS, Koilkonda RD, Chou TH, Porciatti V, Chiodo V (2012). Mutant NADH dehydrogenase subunit 4 gene delivery to mitochondria by targeting sequence-modified adeno-associated virus induces visual loss and optic atrophy in mice. Mol Vis.

[39] Yu H, Koilkonda RD, Chou TH, Porciatti V, Ozdemir SS, Chiodo V (2012). Gene delivery to mitochondria by targeting modified adenoassociated virus suppresses Leber’s hereditary optic neuropathy in a mouse model. Proc Natl Acad Sci U S A.

[40] Liu Y, Eastwood JD, Alba DE, Velmurugan S, Sun N, Porciatti V (2022). Gene therapy restores mitochondrial function and protects retinal ganglion cells in optic neuropathy induced by a mito-targeted mutant ND1 gene. Gene Ther.

[41] Amore G, Romagnoli M, Carbonelli M, Barboni P, Carelli V, La Morgia C (2021). Therapeutic options in hereditary optic neuropathies. Drugs.

[42] Jackson CB, Turnbull DM, Minczuk M, Gammage PA (2020). Therapeutic manipulation of mtDNA heteroplasmy: a shifting perspective. Trends Mol Med.

[43] Gammage PA, Moraes CT, Minczuk M (2018). Mitochondrial genome engineering: the revolution may not be CRISPR-ized. Trends Genet.

[44] Bacman SR, Gammage PA, Minczuk M, Moraes CT (2020). Manipulation of mitochondrial genes and mtDNA heteroplasmy. Methods Cell Biol.

[45] Hirano M, Emmanuele V, Quinzii CM (2018). Emerging therapies for mitochondrial diseases. Essays Biochem.

[46] Reddy P, Ocampo A, Suzuki K, Luo J, Bacman SR, Williams SL (2015). Selective elimination of mitochondrial mutations in the germline by genome editing. Cell.

[47] Mok BY, de Moraes MH, Zeng J, Bosch DE, Kotrys AV, Raguram A (2020). A bacterial cytidine deaminase toxin enables CRISPR-free mitochondrial base editing. Nature.

[48] Mok BY, Kotrys AV, Raguram A, Huang TP, Mootha VK, Liu DR (2022). CRISPR-free base editors with enhanced activity and expanded targeting scope in mitochondrial and nuclear DNA. Nat Biotechnol.

[49] Lim K, Cho SI, Kim JS (2022). Nuclear and mitochondrial DNA editing in human cells with zinc finger deaminases. Nat Commun.

[50] Newman NJ, Yu-Wai-Man P, Biousse V, Carelli V (2022). Understanding the molecular basis and pathogenesis of hereditary optic neuropathies: towards improved diagnosis and management. Lancet Neurol.

[51] Nevinitsina T, Sheremet N, Andreeva N, Zhorzholadze N, Ronzina I, Lyamzaev K (2022). Effect of ophthalmic mitochondrial reactive oxygen species scavenger Visomitin® on visual acuity of patients diagnosed with Leber hereditary optic neuropathy: findings of an observational clinical study. Invest Ophthalmol Vis Sci.

[52] Newman NJ, Biousse V, David R, Bhatti MT, Hamilton SR, Farris BK (2005). Prophylaxis for second eye involvement in leber hereditary optic neuropathy: an open-labeled, nonrandomized multicenter trial of topical brimonidine purite. Am J Ophthalmol.

[53] Leruez S, Verny C, Bonneau D, Procaccio V, Lenaers G, Amati-Bonneau P (2018). Cyclosporine A does not prevent second-eye involvement in Leber’s hereditary optic neuropathy. Orphanet J Rare Dis.

[54] Karanjia R, Coupland SG, Garcia M, Sadun AA (2019). Elamipretide (MTP-131) Topical ophthalmic solution for the treatment of Leber’s hereditary optic neuropathy. Invest Ophthalmol Vis Sci.

[55] Yu-Wai-Man P, Soiferman D, Moore DG, Burte F, Saada A (2017). Evaluating the therapeutic potential of idebenone and related quinone analogues in Leber hereditary optic neuropathy. Mitochondrion.

[56] Giorgio V, Petronilli V, Ghelli A, Carelli V, Rugolo M, Lenaz G (2012). The effects of idebenone on mitochondrial bioenergetics. Biochim Biophys Acta.

[57] Klopstock T, Yu-Wai-Man P, Dimitriadis K, Rouleau J, Heck S, Bailie M (2011). A randomized placebo-controlled trial of idebenone in Leber’s hereditary optic neuropathy. Brain.

[58] Klopstock T, Metz G, Yu-Wai-Man P, Buchner B, Gallenmuller C, Bailie M (2013). Persistence of the treatment effect of idebenone in Leber’s hereditary optic neuropathy. Brain.

[59] Carelli V, La Morgia C, Valentino ML, Rizzo G, Carbonelli M, De Negri AM (2011). Idebenone treatment in Leber’s hereditary optic neuropathy. Brain.

[60] Catarino CB, von Livonius B, Priglinger C, Banik R, Matloob S, Tamhankar MA (2020). Real-world clinical experience with idebenone in the treatment of Leber hereditary optic neuropathy. J Neuroophthalmol.

[61] Committee for Medicinal Products for Human Use (CHMP). Raxone: EPAR - Public assessment report [Internet]. London, United Kingdom: European Medicines Agency; 25 June 2015 [accessed 5 May 2022]. Available from: https://www.ema.europa.eu/en/documents/assessment-report/raxone-epar-public-assessment-report_en.pdf

[62] Klopstock T, Tomasso L, Llòria X (2022). Long-term efficacy and safety of idebenone in patients with LHON in the subacute/dynamic phase: results from the LEROS study. Invest Ophthalmol Vis Sci.

[63] Llòria X, Tomasso L, Klopstock T (2022). Long-term efficacy and safety of idebenone in patients with LHON in the chronic phase: results from the LEROS study. Invest Ophthalmol Vis Sci.

[64] Carelli V, Carbonelli M, de Coo IF, Kawasaki A, Klopstock T, Lagreze WA (2017). International consensus statement on the clinical and therapeutic management of Leber hereditary optic neuropathy. J Neuroophthalmol.

[65] Pemp B, Kircher K, Reitner A (2019). Visual function in chronic Leber’s hereditary optic neuropathy during idebenone treatment initiated 5 to 50 years after onset. Graefes Arch Clin Exp Ophthalmol.

[66] Enns GM, Cohen BH (2017). Clinical trials in mitochondrial disease: an update on EPI-743 and RP103. J Inborn Errors Metab Screen.

[67] Shrader WD, Amagata A, Barnes A, Enns GM, Hinman A, Jankowski O (2011). Alpha-tocotrienol quinone modulates oxidative stress response and the biochemistry of aging. Bioorg Med Chem Lett.

[68] Sadun AA, Chicani CF, Ross-Cisneros FN, Barboni P, Thoolen M, Shrader WD (2012). Effect of EPI-743 on the clinical course of the mitochondrial disease Leber hereditary optic neuropathy. Arch Neurol.

[69] Karanjia R, Chu ER, Rockwell S, Klein M, Miller G, Chicani F (2015). Leber’s hereditary optic neuropathy (LHON) open label clinical study for EPI-743: 2015 update. Invest Ophthalmol Vis Sci.

[70] Chicani CF, Chu ER, Miller G, Kelman SE, Sadun AA (2013). Comparing EPI-743 treatment in siblings with Leber’s hereditary optic neuropathy mt14484 mutation. Can J Ophthalmol.

[71] Chadderton N, Palfi A, Millington-Ward S, Gobbo O, Overlack N, Carrigan M (2013). Intravitreal delivery of AAV-NDI1 provides functional benefit in a murine model of Leber hereditary optic neuropathy. Eur J Hum Genet.

[72] Marella M, Seo BB, Thomas BB, Matsuno-Yagi A, Yagi T (2010). Successful amelioration of mitochondrial optic neuropathy using the yeast NDI1 gene in a rat animal model. PLoS ONE.

[73] Qi X, Sun L, Hauswirth WW, Lewin AS, Guy J (2007). Use of mitochondrial antioxidant defenses for rescue of cells with a Leber hereditary optic neuropathy-causing mutation. Arch Ophthalmol.

[74] Liang KJ, Woodard KT, Weaver MA, Gaylor JP, Weiss ER, Samulski RJ (2017). AAV-Nrf2 promotes protection and recovery in animal models of oxidative stress. Mol Ther.

[75] Osborne A, Khatib TZ, Songra L, Barber AC, Hall K, Kong GYX (2018). Neuroprotection of retinal ganglion cells by a novel gene therapy construct that achieves sustained enhancement of brain-derived neurotrophic factor/tropomyosin-related kinase receptor-B signaling. Cell Death Dis.

[76] Khatib TZ, Osborne A, Yang S, Ali Z, Jia W, Manyakin I (2021). Receptor-ligand supplementation via a self-cleaving 2A peptide-based gene therapy promotes CNS axonal transport with functional recovery. Sci Adv..

[77] Whitehead M, Osborne A, Yu-Wai-Man P, Martin K (2021). Humoral immune responses to AAV gene therapy in the ocular compartment. Biol Rev Camb Philos Soc.

